# Protocol: Complementarity between informal care and formal care to adults: Knowledge mapping through a scoping review of the literature

**DOI:** 10.1002/cl2.70004

**Published:** 2024-12-02

**Authors:** Marta Osório de Matos, Elzbieta Bobrowicz‐Campos, Rosa Silva, Francisca Castanheira, Diana Santos

**Affiliations:** ^1^ Iscte – Instituto Universitário de Lisboa (Iscte‐IUL) Lisbon Portugal; ^2^ Centro de Investigação e Intervenção Social (Cis‐Iscte) Lisbon Portugal; ^3^ Nursing School of Porto (ESEP) Porto Portugal; ^4^ Center for Health Technology and Services Research (CINTESIS@RISE) University of Porto Porto Portugal; ^5^ Portugal Centre for Evidence Based Practice: A JBI Centre of Excellence, Health Sciences Research Unit: Nursing, Nursing School of Coimbra Coimbra Portugal; ^6^ Hospitais da Universidade de Coimbra, Unidade Local de Saúde de Coimbra, EPE Coimbra Portugal; ^7^ Unidade de Investigação em Ciências da Saúde: Enfermagem (UICISA: E), Escola Superior de Enfermagem de Coimbra (ESEnfC) Coimbra Portugal

**Keywords:** complementarity, formal care, informal caregivers

## Abstract

This is the protocol for a Campbell systematic review. The aim of this scoping review is to map, describe and characterize the available evidence on the role that formal care might have in complementing informal caregivers role – romantic partners, parallel family members (e.g., [step]siblings, cousins or in‐laws) or descendants (e.g., the care‐receivers are [step]parents, [step]aunts/uncles, [step]grandparents or in‐laws) – when providing care to adults with acquired diseases (physical or neurologic) in domestic settings and will set the ground for future research on this topic. The scoping review questions are the following: (i) What is the existing literature on the complementarity between formal and informal care? (ii) Which are the types of formal cares' services/interventions that have been described in the literature as complementary to informal care, provided to the informal caregiver and/or to the adult being cared for? (iii) Which outcomes have been assessed in the caregiver's physical, psychological, and social health domains, and how have they been measured?

## 1 BACKGROUND

Caring for a loved one can be a rewarding endeavor, experienced with satisfaction, reciprocity, and closeness (e.g., Wilkins et al., 2020). However, providing informal care (i.e., unpaid care provided by family, friends, and/or neighbors to someone in need) is also a demanding venture (e.g., Gérain & Zech, 2019; Zarit & Savla, 2016). Indeed, long‐term care provision to adults is known to rely heavily on informal caregivers, especially on spouses and adult children (Spasova, et al., 2018; Zigante, 2018), with detrimental physical (e.g., chronic illnesses, musculoskeletal comorbidities), psychological (e.g., depression, sleep disturbances, burnout), and social (e.g., involuntary social isolation) effects of being an informal caregiver of adult relatives (see Schulz et al., 2020 for a review). As one progresses through the life cycle the probability of accumulating health problems increases, increasing the need to provide/receive care, mostly of an informal nature (Bauer et al., 2018; Calvano, 2013). Conversely, due to sociodemographic changes, individuals' needs are expected to outgrow families' ability to support their significant ones, when in need. Therefore, formal care (i.e., paid/contracted services provided by an institution or trained individuals) has become a growing area of support (McNeil, 2014), that might complement the care provided by informal sources. Also, from a systemic perspective, regarding the shortage in formal care services, more research is needed on partnerships for care (World Health Organization: WHO, 2023).

### 1.1 Complementarity between formal and informal care

The concept of complementarity describes the simultaneous use of professionalized (formal care) and informal sources of support to provide care in an informal setting (e.g., home). Several theoretical models have framed the relationship between formal and informal care. Pinquart and Sörensen (2002) provided an important review of models on complementarity between formal and informal care. On one hand, complementarity can be seen as mutual exclusive or as substitution, as one excluding the other (the Hierarchical Compensatory Model [Cantor, 1979] and the Substitution Model [Greene, 1983]). Another set of theories proposes that formal and informal care may coexist and supplement each other when caregiving demands exceed the informal caregiver's capability or availability (Task‐specificity Model [Litwak, 1985]) describes that the different tasks of care provision are dependent on the type of relationship and its structure. Finally, another model incorporates both compensatory and supplementary functions of formal care (The Complementary Model [Chappell & Blandford, 1991; Chappell, 1985]), suggesting that formal care assists the informal network when the latter cannot meet the care needs. Indeed, research has shown that informal and formal support sources can have complementary and supplementary perspectives on the formal‐informal care interface; and community‐based formal care does not substitute for family assistance (Davey & Patsios, 1999). Also, Penning and Keating's (2000) found that formal support is not used to displace or substitute for informal care but, rather, formal services tend to be used to supplement and complement the care provided by the informal network – in community as in residential care settings.

### 1.2 Research gap

Several studies have addressed the correlates of using both types of social support, namely family characteristics that explain contracting formal care (e.g., respite, home care; Aung et al., 2021; Chappell & Blandford, 1991; Firgo et al., 2020; Sun et al., 2021). However, to the best of our knowledge, there is a lack of reviews on the concept of complementarity between formal and informal care. Moreover, the literature that addresses the concomitant provision of formal and informal care to adults still lacks in the operationalization of formal care, not always being clear what kind of formal care is being studied, and due to the lack of investigation on the effects of formal care on informal caregivers' health outcomes limit the possibility of conducting an effectiveness review. In addition, some studies that have explored the association between using formal care and informal caregivers' health outcomes have been retrieving inconsistent results which calls for a mapping of the literature on which is the available evidence and how research has been conducted.

In sum, the present study aims to provide a deep understanding of the available evidence on the role that formal support might have in complementing the role of informal caregivers when providing care to adults with acquired diseases (physical or neurologic) in domestic settings. It will provide a comprehensive and solid groundwork on the studied types of services/interventions that fall under the umbrella of formal care that have been used as complementary to informal caregiving.

A preliminary search, conducted in MEDLINE via PubMed, the Cochrane Database of Systematic Reviews, and JBI *Evidence Synthesis, confirmed that there are n*o current or in‐progress systematic reviews or scoping reviews on the topic *in question*.

## 2 OBJECTIVES

The aim of this scoping review is to map, describe and characterize the available evidence on the role that formal care might have in complementing informal caregivers role – romantic partners, parallel family members (e.g., [step]siblings, cousins or in‐laws) or descendants (e.g., the care‐receivers are [step]parents, [step]aunts/uncles, [step]grandparents or in‐laws) – when providing care to adults with acquired diseases (physical or neurologic) in domestic settings and will set the ground for future research on this topic. The scoping review questions are the following:

(i) What is the existing literature on the complementarity between formal and informal care?
(ii) Which are the types of formal cares' services/interventions that have been described in the literature as complementary to informal care, provided to the informal caregiver and/or to the adult being cared for?
(iii) Which outcomes have been assessed in the caregiver's physical, psychological, and social health domains, and how have they been measured?

## 3 METHODS

This study is being conducted according to the methodological framework for scoping review proposed by the JBI (Peters et al., 2020) and in line with the Preferred Reporting Items for Systematic Reviews and Meta‐Analyses Extension for Scoping Reviews (PRISMA‐ScR; Tricco et al., 2018). The inclusion criteria are defined according to Population, Concept, and Context as presented below.

### 3.1 Inclusion criteria

#### 3.1.1 Population

This scoping review will consider studies focusing on adults who are unpaid informal caregivers to their relatives with one or more acquired chronic diseases (physical or neurological), that: (a) cause progressive or prolonged physical impairment; and/or (b) significantly interfere with instrumental and/or basic activities of daily living.

Inclusion criteria regarding caregiver relationship with the care‐receiver will be the existence of (a) romantic relationship (e.g., spouse, boy/girlfriend) or; (b) a parallel relationship (e.g., the care‐receivers are [step]siblings, cousins, or in‐laws); or (c) descending relationship with the care‐receivers (e.g., the care‐receivers are [step]parents, [step]aunts/uncles, [step]grandparents or in‐laws). Therefore, studies that focus solely on caregivers that are parents will be excluded from the review. Whenever a study has only a sub‐set of the eligible caregiver relationship with the care‐receivers' (as described above), only the data related to the above‐identified participants will be extracted. Regarding age, both (caregiver and receiver) must be adults (18 years or older).

Concerning the care‐receiver characterization, exclusion criteria are related to psychiatric/psychologic illness, addiction (related to drugs, gambling, sex, etc.), or if bereavement/grief are the main reasons for receiving informal care. To avoid any confounding effects, we will also consider as not eligible for inclusion studies that focus on caregivers of war veterans, pregnant or postpartum persons, or transgender persons – due to the time frame and/or the specificity of the conditions underlying the need for providing informal care.

The included studies will include data with caregivers that are providing care to persons that are alive at the time of data collection and, therefore, studies with bereaved caregivers will be excluded.

#### 3.1.2 Concept

The concept of interest is the complementarity between formal and informal care based on the conceptual framework of the Complementary Model which considers both the compensatory and supplementary functions of formal care, which comes in to help the informal network when care recipients are in need and critical elements of the informal network are missing (Chappell & Blandford, 1991; Chappell, 1985). The studies to be included must report the utilization of formal care (i.e., paid/contracted services provided by an institution or trained individuals), regularly and/or sporadically, provided in‐person or using technology to informal caregiver and/or care‐receiver, that may include medical, social, or community components or a combination of these. To be included in the review, the study must at least describe the type of service provided or the professional involved in care provision, or care provision duration or intensity (e.g., 2 h/day or part of the day) or frequency (e.g., 3 days/week). Also, regarding the concept of interest, formal care must be complementary therefore provided for part of the day (e.g., day‐care center) or during a specified timeframe (e.g., respite). Articles that refer only to formal care or only to informal care will not be considered in this review. Finally, the studies to be included have to report/measure at least one domain of informal caregiver health (physical, psychological, or social).

#### 3.1.3 Context

This review will consider studies conducted in any geographical, sociocultural, or socioeconomic settings. Only studies in which informal care takes place in informal settings (not hospitals, hospices, or other similar contexts) will be included.

#### 3.1.4 Type of evidence sources

The sources of interest will include primary studies of a qualitative, quantitative, or mixed‐method nature. Relevant gray literature will also be considered for inclusion. Review policy documents, opinion papers, and lay literature will be excluded as they do not report the results of original, empirical investigation, as well as secondary studies as they may not provide all the relevant details of the research conducted.

Articles written in English, Portuguese, Spanish, French, and Polish will be considered for inclusion, with no time restriction regarding the period of their posting in the database, which will allow a comprehensive view of the topic under study.

### 3.2 Search strategy

The search strategy will aim to find both published and unpublished studies, through a three‐step approach:

1. Preliminary searches of MEDLINE via PubMed and CINAHL Complete via EBSCO to identify relevant text words and index terms used to describe the articles on the topic in question.
2. Using all identified text words and index terms, that resulted from the preliminary search, to comprehensively search the included electronic databases.
3. Hand search of the reference lists of all included articles and table contents search of relevant journals, to identify additional studies that meet inclusion criteria.
4. Experts on the topic and international organizations will be contacted for unpublished or ongoing studies, through their public contacts.

The search for published studies will be conducted in the following databases: CINAHL Complete via EBSCO, MEDLINE via EBSCO, SciELO, Web of Science, Scopus, PsycINFO, APA PsychArticles, and Psychology and Behavioral Science Collection. The selection of databases considered the need to provide complete and relevant multidisciplinary coverage. These databases are critical in retrieving research across disciplinary fields relevant for this topic.

The search for unpublished studies will be conducted in the following databases: Open Dissertations, OpenGrey, Repositório Científico de Acesso Aberto de Portugal (RCAAP, Portuguese Open Access Scientific Repository), and Catálogo de teses e Dissertações da CAPES [CAPES theses and dissertations catalog]. These databases allow multidisciplinary coverage. Furthermore, these databases included sources such as research reports, dissertations and thesis which, despite collecting relevant evidence, are often outside of commercial publishing. Considering unpublished literature in the review allows for minimization of the impact of publication bias, potentially complementing the findings from the published sources (Aromataris & Munn, 2020; Lefebvre et al., 2023).

Initial keywords include “care*,” “caregiver,” “care‐dyads,” “family,” “relative,” “formal care,” “formal support,” “social support,” “community support,” “informal support,” “family support,” “informal care.” During the search process, various terminologies and spellings of the keywords will be taken into consideration as they might affect the identification of relevant studies. The search strings will also be optimized for each included database and information source. A final list of search terms and combinations will be agreed between review team members. A detailed example of the proposed search strategy, including all keywords and MeSH terms, can be found in Appendix SI.

To ensure that the review is up to date, if the review is published after 12 months from the initial search date databases will be searched again to incorporate new studies that fit the inclusion criteria (Tricco et al., 2018).

### 3.3 Evidence selection

Once the search is completed, citations will be imported into Rayyan (Qatar Computing Research Institute, Doha, Qatar), designed to facilitate the organization and management of literature reviews (Ouzzani et al., 2016). The duplicates will be removed before screening, afterwards two independent reviewers will screen titles and abstracts according to the eligibility criteria regarding population, concept, and context. Then, the full texts of potentially relevant articles will be retrieved and assessed in detail, by two independent reviewers, according to the full‐text screening guide in Appendix SII. Full‐text articles that do not meet inclusion criteria will be removed and reasons for exclusion will be reported.

Any discrepancies between reviewers, at any point, will be resolved through discussion and consensus. Whenever necessary a third reviewer will be consulted to solve the discrepancies. The results of the study selection process will be reported in full in the scoping review and presented in a PRISMA‐ScR flow diagram (Page et al., 2022).

### 3.4 Data extraction

Data extraction will be done by two reviewers independently, in a systematic and descriptive manner, to address the research aim and questions. A data extraction form was developed and piloted by the research team – Appendix SIII. Data extracted will include details describing each study and referring to participants, concepts, context, and key findings relevant to the review questions, such as authors, study objective, design, data collection, population, description of formal care, description of caregivers' outcomes (Pollock et al., 2023). In case of missing or unclear information, the authors of the primary studies will be contacted to provide additional data. Discrepancies will be resolved by discussion until a consensus is reached.

A draft data charting tool can be found in appendix, that was piloted between three team members. To ensure the accuracy of the data extraction tool and verify the level of agreement between reviewers, an initial pilot test of three to five articles will be conducted. Based on these test results, the data extraction tool will be revised and modified if necessary. Modifications will be detailed in the scoping review. Data extracting will be done in duplicate, which reduces both the risk of making mistakes and the possibility that data selection is influenced by a single person's biases.

### 3.5 Reporting the results

The extracted data will be presented in diagrammatic or tabular format, as presented in Appendix SII. However, other formats may be considered after data analysis if they allow better representation of the results. A narrative summary will accompany the tabulated and/or charted results and will describe how the results relate to the review's objective and questions. The aim of mapping the data is to identify, characterize and summarize the research evidence on the topics under study. The elements of the Population, Concept and Context inclusion criteria guide the way in which the data will be mapped.

## CONTRIBUTIONS OF AUTHORS

MM, EBC, and FC developed the research questions, objectives of the study, and study design. MM and EBC planned the methodology of the study. MM, EBC, RS and DS developed the search strategy. EBC, RS, and DS performed the preliminary search. MM and DS developed the data charting form. All authors approved of the final text of the protocol sent to be published.

## DECLARATIONS OF INTEREST

The authors declare that they have no competing interests.

## SOURCES OF SUPPORT

### Internal sources

- No sources of support provided.

### External sources

- No sources of support provided.

## Supporting information

Supporting information.

Supporting information.
