# Supplementary material for: Protocol: Complementarity between informal care and formal care to adults: Knowledge mapping through a scoping review of the literature
Source: Campbell Syst Rev. 2024 Dec 2;20(4):e70004. doi: 10.1002/cl2.70004 (PMC11609753; doi:10.1002/cl2.70004)
Supplement: Supplementary file 1 — Supporting information. [file CL2-20-e70004-s001.docx]

# Appendices

# Appendix I: Example of Search Strategy

**MEDLINE via EBSCO**

| S1 | TI (care* OR caregiver* OR “care giver” OR “care givers” OR care-dyads OR famil* OR relative* OR spouse* OR son OR sons OR daughter*) |
| --- | --- |
| S2 | AB (care* OR caregiver* OR “care giver” OR “care givers” OR care-dyads OR famil* OR relative* OR spouse* OR son OR sons OR daughter*) |
| S3 | MH (Caregivers) |
| S4 | S1 OR S2 OR S3 |
| S5 | TI (“formal care” OR “formal support” OR “nursing care” OR “social care” OR “social support” OR “home care” OR “paid care” OR “community care” OR “community support”) |
| S6 | AB (“formal care” OR “formal support” OR “nursing care” OR “social care” OR “social support” OR “home care” OR “paid care” OR “community care” OR “community support”) |
| S7 | MH (Home Care Services OR Nursing Care OR Formal Assistance OR Adult Day Care Centers OR Home Care Services, Hospital-Based OR Day Care, Medical OR Home Health Nursing OR Psychosocial Support Systems OR Social Support OR Community Support OR Patient Care OR Respite Care) |
| S8 | S5 OR S6 OR S7 |
| S9 | TI (“informal support” OR “family support” OR “informal care”) |
| S10 | AB (“informal support” OR “family support” OR “informal care”) |
| S11 | MH (Family Support OR Informal Care OR Home Care Agencies OR Home Nursing) |
| S12 | S9 OR S10 OR S11 |
| S13 | S4 AND S8 AND S12 |

# Appendix II: Full text screening guide

| If YES, then INCLUDE | |
| --- | --- |
| 1. Article characteristics | - 1. The article is a full-text’ research study published after a peer-review process (regardless of the methodological strategy or statistical method used). |
|  | 1.2. The article is written in English **OR** Portuguese **OR** Spanish **OR** French **OR** Polish |
|  | - 1. Study is empirical **AND** not a review. |

| If YES, then INCLUDE | |
| --- | --- |
| 1. Study Methodology | 2.1. Sample is constituted partially **OR** completely by adult Informal caregivers (18 years old or more)^(i.e., unpaid care provided by people refered in 2.2)^? |
|  | 2.2. Caregiver sample is composed by:  a) romantic relationship (e.g., spouse, boy/girlfriend)  AND/OR;  b) a parallel relationship (e.g., the care-receivers are [step]siblings, cousins, or in-laws);  AND/OR  c) descending relationship with the care-receivers (e.g., the care-receivers are [step]parents, [step]aunts/uncles, [step]grandparents or in-laws).  Whenever a study has only a sub-set of the eligible caregiver relationship with the care-receiver, only the data related to the above-identified participants will be extracted. |
|  | - 1. The study is **NOT** a retrospective study on informal caregiving to people (care-receivers) that have died at the time of data collection? |
|  | - 1. In quantitative studies, are physical ^(e.g., chronic illnesses, musculoskeletal comorbidities)^ **OR** psychological ^(e.g., depression, sleep disturbances, burnout)^ **OR** and social health ^(e.g., loneliness, involuntary social isolation, social integration, and participation)^ outcomes of informal caregivers measured? |
|  | - 1. In qualitative studies, are physical ^(e.g., chronic illnesses, musculoskeletal comorbidities)^ **OR** psychological ^(e.g., depression, sleep disturbances, burnout)^ **OR** and social health ^(e.g., loneliness, involuntary social isolation, social integration, and participation)^ outcomes are considered as sample characteristic and/or unit of analysis? |
|  | - 1. In quantitative studies, is **formal care**, defined as paid/contracted/provided services provided by an institution or trained individuals ^(i.e., may include medical, social, or community services or a combination of these OR may be an intervention)^,measured/operationalized **OR** mentioned has being provided to Informal Caregiver **OR** Care-Receiver?   Whenever a study has only a sub-set of participants that receive formal care, only the data related to this sub-sample will be extracted. |
|  | - 1. In qualitative studies, is **formal care**, defined as paid/contracted services provided by an institution or trained individuals ^(i.e., may include medical, social, or community services or a combination of these OR may be an intervention)^, considered as sample characteristic and/or unit of analysis?   Whenever a study has only a sub-set of participants that receive formal care, only the data related to this sub-sample will be extracted. |
|  | - 1. Formal care can be provided in person **OR** using technology. |
|  | - 1. Formal support is described/operationalized by stating: - Type of service/intervention provided (e.g. daycare center, respite, phone call) **OR** Professional/Caregiver.   **AND/OR**   - Duration **OR** Intensity (e.g., 2 hrs/day or part of day) **OR** frequency (e.g., 3 days/week). |
|  | - 1. Formal care assists caregiver/care-receiver for part of the day (day-care center) **OR** a portion of time (is temporary, like respite). |
|  | - 1. Informal care provision takes place in domestic settings (**NOT** in nursing homes, hospitals, hospices, or other similar contexts)? |

| If YES, then INCLUDE | |
| --- | --- |
| 1. Care-receiver characteristics | 3.1. Is an adult. |
|  | 3.2. Care-receiver has one **OR** more acquired (not born with) chronic (not acute) disease (physical **OR** neurologic) that causes progressive **OR** prolonged physical impairment  **AND/OR**  has a chronic disease that significantly interferes with instrumental **AND/OR** basic activities of daily living |
|  | 3.3. Has NOT psychiatric/psychologic illness |
|  | 3.4. Has NOT neurological diseases with onset in childhood |
|  | 3.5. Has NOT Addiction ^(e.g. drugs, gambling, sex, smoking)^ |
|  | 3.6. Is NOT in grief/bereavement |
|  | 3.7. Is NOT a war veteran |
|  | 3.8. Is NOT a pregnant person NOR in post-partum |
|  | 3.9. Is NOT a transgender person |

# Appendix III: Data charting form

Table 1. Description of the studies – Research question 1: What is the existing literature on the of complementarity between formal and informal care?

| **Description of the studies** | | | | | | | | |
| --- | --- | --- | --- | --- | --- | --- | --- | --- |
| Authors/Year | Study objective | Funding | Country | Methodology | Design | Data collection procedures | Participants characteristics | Description of informal caregiving (e.g, Duration/intensity of care, living arrangements) |
|  |  |  |  |  |  |  |  |  |

Table 2. Description of formal care – Research question 2: Which are the types of formal care’ services/interventions that have been described in the literature as complementary to informal care, provided to the informal caregiver and/or to the adult being cared for?

| **Description of formal care** | | | | | | |
| --- | --- | --- | --- | --- | --- | --- |
| Authors/Year | Type | Provided to | Professional | Duration (months,years) | Frequency (daily or weekly) | Other information |
|  |  |  |  |  |  |  |

Table 3. Description of caregivers’ outcomes – Research question 3: Which outcomes have been assessed in the caregiver's physical, psychological, and social health domains, and how have they been measured?

| **Outcomes assessed in the domains of physical, psychological, and social health of the caregiver** | | |
| --- | --- | --- |
| Authors/Year | Description of outcomes | Assessment method/Instruments |
|  |  |  |
